# An Innovative In Vivo Model for CAR-T-Cell Therapy Development: Efficacy Evaluation of CD19-Targeting CAR-T Cells on Human Lymphoma, Using the Chicken CAM Assay

**DOI:** 10.3390/ijms27020795

**Published:** 2026-01-13

**Authors:** Yan Wang, Chloé Prunier, Inna Menkova, Xavier Rousset, Anthony Lucas, Tobias Abel, Jean Viallet

**Affiliations:** 1 Inovotion, 38700 La Tronche, France; 2 Allogenica, 69008 Lyon, France; 3 Institute for Advanced Biosciences, INSERM U1209, CNRS UMR5309, Université Grenoble Alpes, 38000 Grenoble, France

**Keywords:** chicken chorioallantoic membrane (CAM), CAR-T, CD19, Raji lymphoma, new approach methodologies (NAMs), 3Rs

## Abstract

Chimeric antigen receptor (CAR)-T-cell therapy is a revolutionary approach in immunotherapy that has shown remarkable success in the treatment of blood cancers. Many preclinical studies are currently underway worldwide to extend the CAR-T-cell therapy benefits to a broad spectrum of cancers, using rodent models. Alternative in vivo platforms are essential for overcoming the drawbacks associated with rodent models, including immunodeficiency in humanized models, ethical concerns, extended time requirements, and cost. In this work, we used the chicken chorioallantoic membrane (CAM) assay to evaluate the in vivo efficacy of cluster-of-differentiation 19 (CD19)-targeting CAR-T cells expressing a second-generation CAR construct against human lymphoma derived from the Raji cell line. Our results confirm the efficacy of selected CAR-T cells on tumor growth, metastasis, and angiogenesis. Further, the chicken embryo has an intrinsic active immune system. Therefore, the dialog between CAR-T cells and endogenous immune cells, as well as their participation in the tumor challenge, has also been studied. In conclusion, our study demonstrates that the chicken CAM assay provides a relevant in vivo, 3Rs (Replacement, Reduction and Refinement)-compliant new approach methodology (NAM), which is well-suited for the current needs of preclinical research on CAR-T-cell therapy.

## 1. Introduction

Immunotherapy is now regarded as the ‘fifth pillar’ of cancer treatment, alongside surgery, radiotherapy, chemotherapy, and other targeted therapies. Of various cancer immunotherapy strategies that have been developed, T-cell-based therapies are among the most promising methods, with chimeric antigen receptor-T (CAR-T)-cell therapy being a prototypic treatment [1,2,3]. CAR-T-cell therapy is a revolutionary approach that genetically engineers T cells to recognize antigens expressed by tumor cells and eliminate these cells specifically. Unlike T-cell receptor (TCR) cell therapy, the recognition between chimeric antigen receptor (CAR) and target antigens is major histocompatibility complex (MHC)-independent. Therefore, CAR-T-cell therapy can induce vigorous T-cell activation and powerful anti-tumor responses, which are useful for treating a broad range of cancer patients [4,5].

Since the study of engineered T cells with chimeric molecules was first reported by Zelig Eshhar in 1993 [6], the development of CAR-T cells has advanced across several generations, and notable success has been reported in the treatment of blood cancers [7,8]. A total of six commercially available CAR-T products based on a second-generation CAR construct have now been approved by the FDA to treat B-cell malignancies and multiple myeloma, of which four products are cluster-of-differentiation 19 (CD19)-targeting [9]. Despite encouraging clinical outcomes, CAR-T-cell therapies are still facing several important limitations to broad application, including life-threatening CAR-T-cell-associated toxicities, limited efficacy against solid tumors, inhibition and resistance in B-cell malignancies caused by antigen escape, limited persistence, poor trafficking and tumor infiltration, and an immunosuppressive microenvironment [10].

Many preclinical and clinical trials are ongoing worldwide to improve the efficacy of CAR-T-cell therapies and expand their benefits to a wider range of cancers. For preclinical research, rodents remain the most commonly used in vivo models. However, rodent models present several limitations, such as the immunodeficiency of humanized models, ethical constraints, time requirements, and high cost. Therefore, there is a strong need for an alternative, relevant in vivo model for developing CAR-T-cell therapy. The chicken embryo as an experimental model for human non-cancer biology research has a long and productive history, owing to its accessibility and strong relevance to human physiology [11]. In recent decades, the chicken embryo’s chorioallantoic membrane (CAM) has been widely used in various fields of biomedical research [12,13]. The CAM is a highly vascularized extraembryonic structure that allows the exchange of gases and nutrients to the embryo during the entire period of its development. It also facilitates calcium mobilization from the eggshell, thereby promoting embryonic bone mineralization [14,15]. Owing to its unique tissue composition, visibility, accessibility, and rapid development, the CAM is a potent, alternative preclinical in vivo model used for studies of vasculogenesis and angiogenesis, cancer research, drug development, regenerative medicine, and bioengineering, among others. Since its first application in oncology, reported by Rous and Murphy in 1911, that demonstrated the growth of chicken sarcoma tumors transplanted onto the CAM, this model has proved extremely valuable for human tumor xenografts and cancer research. It offers a platform suitable for studying various aspects of tumor development, such as angiogenesis, proliferation, and metastatic invasion [16,17,18,19], as well as for immune-based studies [20,21,22]. Indeed, the chicken immune system has been well studied and described as simpler yet functionally similar to the human immune system. The chicken embryo model exhibits partial immunity before embryonic-development day (EDD) 9, allowing the grafting of human cells onto the CAM with a low risk of transplant rejection, while gradually building its immunocompetence [23]. This unique feature allows the evaluation of various immuno-oncology drugs in a physiological, immune-reactive in vivo environment. Moreover, it offers significant ethical value by adhering to the ‘Replacement, Reduction, and Refinement’ (3Rs) principles of humane experimental techniques [24,25]. The above-mentioned considerations support the use of the chicken CAM model for the preclinical validation of CAR-T-cell therapy candidates.

This work aims to provide a proof-of-concept to evaluate the relevance of the chicken CAM assay by testing the efficacy of CD19-targeting CAR-T-cell products developed by Allogenica (France) on human lymphoma originating from the Raji cell line. The results could lead to more in-depth analyses of the underlying mechanisms.

## 2. Results

### 2.1. Raji Cells’ In Vitro Characterization and In Ovo Grafting

**Figure 1:**
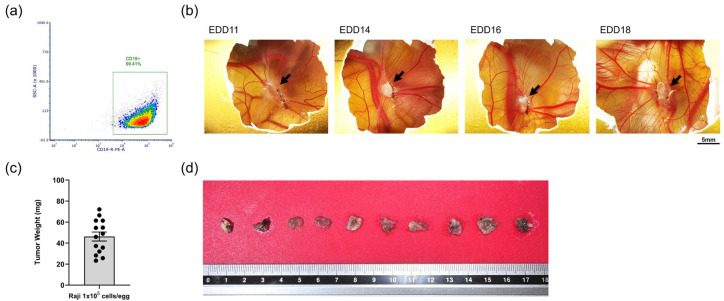
In vitro characterization and in ovo grafting of Raji cells (in ovo data were obtained from one cohort of embryos within a single experimental run): (**a**) Surface expression on CD19 by Raji cells confirmed by flow cytometry; (**b**) representative photos illustrating in ovo growth of tumors derived from the Raji cell line (black arrow points to the tumor; scale bar = 5 mm); (**c**) mean value ± SEM (mg) of tumor weight measured at EDD18 (*n* = 14); (**d**) representative photo of tumors collected at EDD18 and fixed in 4% PFA.

To evaluate the suitability of the Raji tumor model for in ovo drug testing, we assessed target antigen expression in vitro and evaluated tumor-forming ability after in ovo xenografting. Based on flow cytometry analysis, the target antigen—CD19, a surface biomarker for B lymphocytes—was consistently expressed by Raji cells (Figure 1a). In ovo, after Raji cells were grafted at 1 × 10^5^ cells per egg at EDD9, sustained tumor growth without regression was observed in all samples until EDD18 (Figure 1b). The average tumor weight was 53.89 mg ± 3.29 mg SEM (standard error of the mean) (Figure 1c), and all tumors formed in ovo were morphologically similar (Figure 1d). These findings indicate that the model yields consistent tumor growth that is adequate for in ovo drug-testing. Therefore, Raji in ovo xenografting was validated as suitable for evaluating CD19-targeting CAR-T cells’ efficacy.

### 2.2. In Ovo Anti-Tumor Efficacy of CD-19 Targeting CAR-T Cells

**Figure 2:**
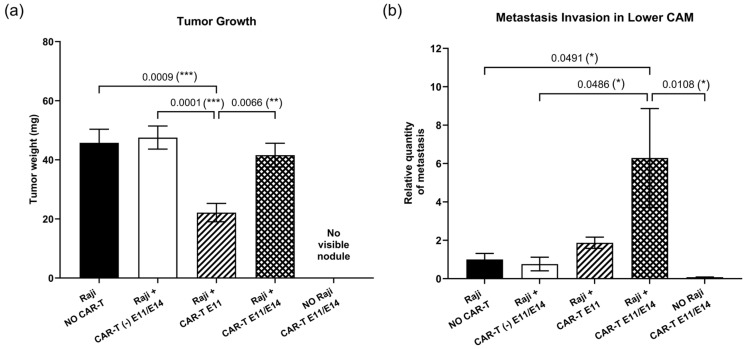
In ovo anti-tumor efficacy of CD19 CAR-T (CD3z-CD28) cells investigated at EDD18 (data were obtained from five cohorts of embryos within a single experimental run): (**a**) Tumor growth evaluation through tumor mass weight (mean value ± SEM) in different groups (*n* = 16–20 per group); (**b**) metastasis evaluation through relative *Alu* sequence expression (mean value ± SEM) in the lower CAM in different groups measured by qPCR, with an arbitrary value of *Alu* expression at 1 for the non-treated (negative control) group (*n* = 8 per group). Statistics were calculated through a one-way ANOVA analysis (with Tukey’s multiple comparison post-tests for all pairwise group comparisons): no stars, no statistical difference (*p* value > 0.05); one star (*), 0.05 ≥ *p* value > 0.01; two stars (**), 0.01 ≥ *p* value > 0.001; three stars (***), 0.001 ≥ *p* value > 0.0001.

To evaluate the anti-tumor efficacy of CAR-T cells in ovo, CD19 CAR-T (CD3z-CD28) cells were first tested. Raji cells were grafted on the upper CAM at EDD9 and CAR-T cells were dosed either once at EDD11 (group “Raji + CAR-T E11” in Figure 2), or twice at EDD11 and EDD14 (group “Raji + CAR-T E11/E14” in Figure 2). To interpret the efficacy of the CAR-T cells, several control groups were included (see in Figure 2): (I) “Raji, NO CAR-T”, in which embryos were grafted with Raji cells but did not receive any treatment, to serve as the negative control; (II) “NO Raji, CAR-T E11/E14”, in which embryos were not grafted with Raji cells at EDD9 but rather administered with CAR-T cells at EDD11 and EDD14—this was used to check if the administration of CAR-T cells could result in nodule formation on the CAM and to reveal the *Alu* signal induced by CAR-T cells in the lower CAM, for properly interpreting Raji tumor growth and metastasis; and (III) “Raji + CAR-T (-) E11/E14”, in which embryos were grafted with Raji cells at EDD9 and treated with the control “CAR-T (-) T cells” expressing only CAR at the cell surface but not the intracellular-activation signaling domain, was used to confirm the importance of the integrity of all functional units (i.e., extracellular CAR, the transmembrane domain and the intracellular-activation domain) and to reflect potential effects attributable to an adaptive immune response against CAR-T cells. First, in terms of tumor growth (Figure 2a), when compared to the negative control “Raji, NO CAR-T” (showing a tumor mean weight of 45.76 mg), a significant tumor growth regression was found in the group treated once with CAR-T cells (“Raji + CAR-T E11”, for a tumor mean weight of 22.15 mg, obtaining a regression of 51.60%, *** *p* = 0.0009), but not in the group treated twice with CAR-T cells (“Raji + CAR-T E11/E14”, the mean weight of 41.59 mg, a regression of 9.11%, *p* = 0.9481). CAR-T (-) cells did not impact the tumor growth (“Raji + CAR-T (-) E11/E14”, the mean weight of 47.53 mg, a progression of 1.04%, *p* = 0.9977), and the administration of CAR-T cells (“NO Raji, CAR-T E11/E14”) did not lead to the formation of nodules on the CAM. Second, regarding metastatic load in the lower CAM (Figure 2b), correlated to the negative control “Raji, NO CAR-T” with an arbitrary value of *Alu* expression at 1, no statistically relevant change was found in the group treated once with CAR-T cells at EDD11 (“Raji + CAR-T E11”, for a relative *Alu* expression value of 1.871, obtaining a progression of 87.1%, *p* = 0.9901), nor in the group treated with control CAR-T cells (“Raji + CAR-T (-) E11/E14”, the relative value of 0.762, a regression of 23.8%, *p* > 0.9999). However, a significant metastasis progression was found in the group treated twice with active CAR-T cells (“Raji + CAR-T E11/E14”, the relative value of 6.288, a progression of 528.8%, * *p* = 0.0491). Meanwhile, CAR-T cells did not contribute to an evident *Alu* signal in the lower CAM when they were administered alone (“NO Raji, CAR-T E11/E14”, a relative quantity of 0.078).

### 2.3. In Ovo CAR-T-Cell Functionality

**Figure 3:**
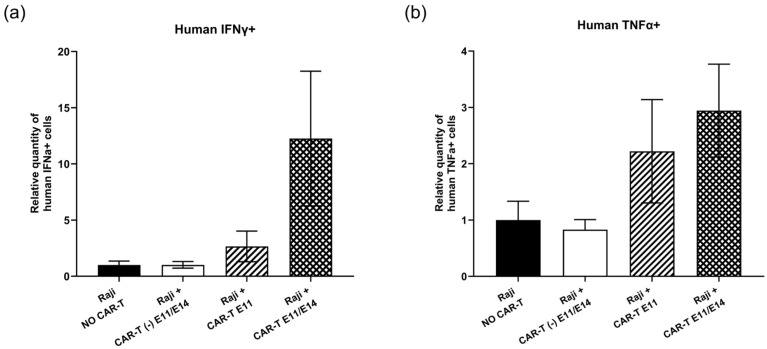
In ovo CD19 CAR-T (CD3z-CD28) cells’ functionality investigated at EDD18 (data were obtained from four cohorts of embryos within a single experimental run): (**a**) Relative quantity (mean value ± SEM) of intra-tumoral human IFNα+ cells in different groups measured by RT-qPCR (*n* = 8 per group); (**b**) relative quantity (mean value ± SEM) of intra-tumoral human TNFα+ cells in different groups measured by RT-qPCR, with an arbitrary value of biomarker expression at 1 for the non-treated (negative control) group (*n* = 8 per group). Statistics were calculated through a one-way ANOVA analysis (with Tukey’s multiple comparison post-tests for all pairwise group comparisons): no stars, no statistical difference (*p* value > 0.05).

Regarding the tumor growth change following CAR-T-cell treatment, the intra-tumoral gene expression of two human markers (interferon gamma (IFNγ) and tumor necrosis factor alpha (TNFα)), which represent CAR-T cells’ functionality, was analyzed at EDD18 by RT-qPCR, to interpret the action of CD19 CAR-T (CD3z-CD28) cells in ovo. First, in terms of human IFNγ gene expression (Figure 3a), when compared to the negative control (“Raji, NO CAR-T”), although not significant, an increase of 12.25-fold was found in the group treated twice with CAR-T cells (“Raji + CAR-T E11/E14”), and an increase of 2.66-fold was detected in the group treated once with CAR-T cells (“Raji + CAR-T E11”), while no evident change was observed in the group treated with control CAR-T cells (“Raji + CAR-T (-) E11/E14”). Second, a similar tendency was detected for intra-tumoral human TNFα gene expression (Figure 3b). A 2.95-fold increase was observed in the “Raji + CAR-T E11/E14” group, while a 2.22-fold increase was observed in the “Raji + CAR-T E11” group, and no evident change was found in the “Raji + CAR-T (-) E11/E14” group.

### 2.4. Endogenous Immune Reaction Induced by CAR-T Cells

**Figure 4:**
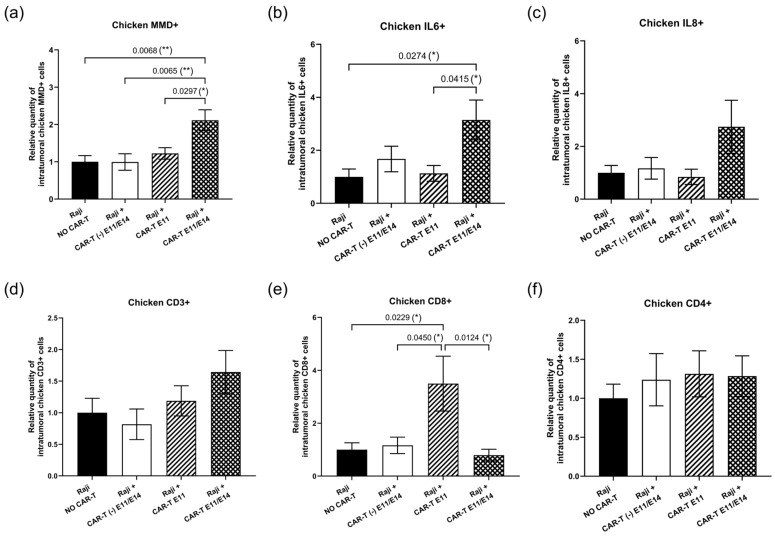
In ovo endogenous immune response triggered by CD19 CAR-T (CD3z-CD28) cells (data were obtained from four cohorts of embryos within a single experimental run): The intra-tumoral expression of different chicken immune markers was investigated at EDD18, based on RT-qPCR analyses, correlated to the non-treated (negative control) group (*n* = 8 per group). The relative expression level (mean value ± SEM) is shown for (**a**) MMD, (**b**) IL-6, (**c**) IL-8, (**d**) CD3, (**e**) CD8, and (**f**) CD4. Statistics were calculated through a one-way ANOVA analysis (with Tukey’s multiple comparison post-tests for all pairwise group comparisons): no stars, no statistical difference (*p* value > 0.05); one star (*), 0.05 ≥ *p* value > 0.01; two stars (**), 0.01 ≥ *p* value > 0.001.

Considering the metastasis progression observed in the group treated twice with CD19 CAR-T (CD3z-CD28) cells (Figure 2b), we investigated the activation of the endogenous immune response triggered by CAR-T cells and the participation of chicken embryo immune cells in the tumor challenge. To this end, the intra-tumoral expression of chicken immune markers was evaluated by RT-qPCR. First, the monocyte-to-macrophage differentiation-associated (MMD) marker was used to measure macrophage activity. Compared to the negative control (“Raji, NO CAR-T”), MMD expression was significantly increased in the group treated twice with CAR-T cells (“Raji + CAR-T E11/E14”), showing an increase of 2.11-fold (** *p* = 0.0068). This increase was also significant when compared to the group treated with control CAR-T cells (“Raji + CAR-T (-) E11/E14”, ** *p* = 0.0065) and the group treated once with CAR-T cells (“Raji + CAR-T E11”, * *p* = 0.0297) (Figure 4a). Second, the expression of two pro-inflammatory factors, interleukin 6 (IL6) and interleukin 8 (IL8), was also investigated in the tumor site (Figure 4b,c). Similar results were obtained for these two markers: upregulation (an increase of 3.15-fold for IL6, and an increase of 2.74-fold for IL8) was found in the “Raji + CAR-T E11/E14” group. For IL6, the upregulation found in the “Raji + CAR-T E11/E14” group was statistically significant when compared to the “Raji, NO CAR-T” (* *p* = 0.0274) group, as well as when compared to the “Raji + CAR-T E11” group (* *p* = 0.0415). Third, CD3 (Figure 4d), CD8 (Figure 4e), and CD4 (Figure 4f) markers were assessed for the presence of chicken T lymphocytes. When compared with the “Raji, NO CAR-T” group, an increased CD3 expression showed an upward trend in the CAR-T treated groups (an increase of 1.19-fold in “Raji + CAR-T E11” and 1.64-fold in “Raji + CAR-T E11/E14”), but not in the control CAR-T (Raji + CAR-T (-) E11/E14) group, where we found a decrease of 0.82-fold. A significantly increased CD8 expression was detected in the “Raji + CAR-T E11” group (a 3.50-fold increase, * *p* = 0.0229), and this increase was also evident when compared to the “Raji + CAR-T (-) E11/E14” group (* *p* = 0.045) and to the “Raji + CAR-T E11/E14” group (* *p* = 0.0124); meanwhile, the expression of the CD4 marker was similar in all groups, no statistically significant change was found.

### 2.5. Tumor Histological Status

**Figure 5:**
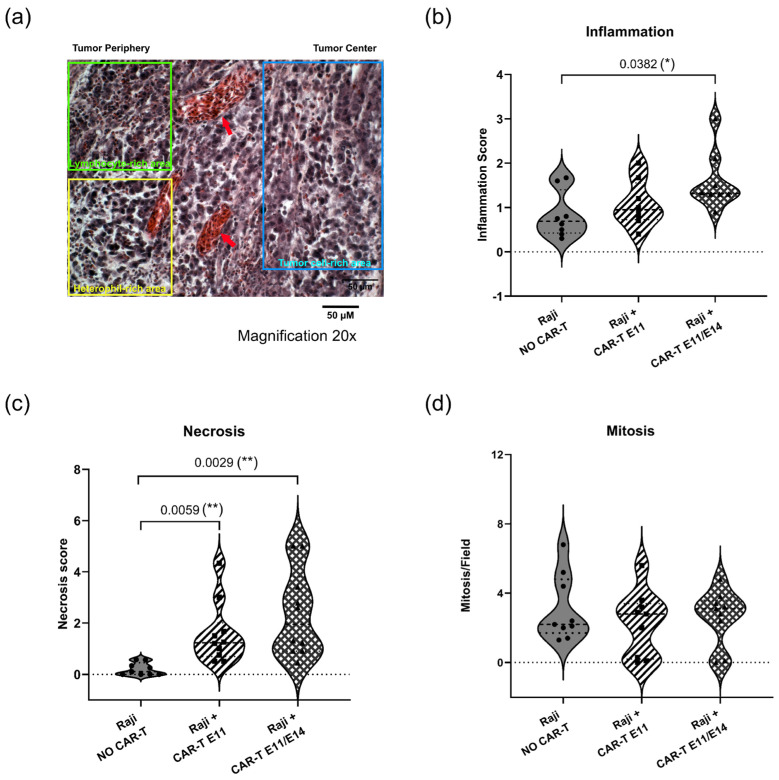
Tumor status analyzed through hematoxylin and eosin (H&E) staining (data were obtained from three cohorts of embryos within a single experimental run). (**a**) shows the representative histology image of Raji tumors developed in ovo (scale bar = 50 μm, 20× magnification): the blue rectangle shows the tumor cell-rich area, the yellow rectangle shows the heterophil-rich area; the green rectangle shows the lymphocyte-rich area; and red arrows point to the vessels. Semi-quantitative analyses using a scoring scale were performed for (**b**) inflammation, (**c**) necrosis, and (**d**) mitosis interpretations. Image analyses were performed on 10 fields per section. Statistics were calculated through a one-way ANOVA analysis (with Tukey’s multiple comparison post-tests for all pairwise group comparisons): no stars, no statistical difference (*p* value > 0.05); one star (*), 0.05 ≥ *p* value > 0.01; two stars (**), 0.01 ≥ *p* value > 0.001.

To further characterize CAR-T-cell effects on tumor growth beyond RT-qPCR analysis, tumor tissues in the negative control (“Raji, NO CAR-T”) and in the groups treated with CD19 CAR-T (CD3z-CD28) (“Raji + CAR-T E11” and “Raji + CAR-T E11/E14”) were analyzed by hematoxylin and eosin (H&E) staining (Figure 5a). Histological parameters such as inflammation (Figure 5b), necrosis (Figure 5c), and mitosis (Figure 5d) were investigated. When compared to the negative control (“Raji, NO CAR-T”), a stronger inflammatory reaction was observed in the group treated twice with CAR-T cells (“Raji + CAR-T E11/E14”, * *p* = 0.0382); more necrotic tissues were found in the “Raji + CAR-T E11” group (** *p* = 0.0059) and the “Raji + CAR-T E11/E14” group (** *p* = 0.0029); meanwhile, a slight decrease in the mitotic index was found in both groups treated with CAR-T cells, even though the change was not statistically significant.

### 2.6. Evaluation of CAR-T-Cell Therapy Efficacy at an Optimized Dose

**Figure 6:**
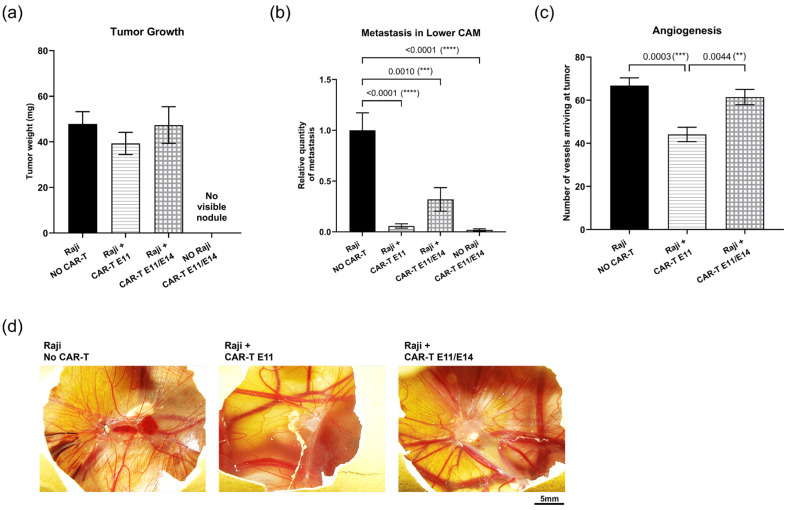
In ovo evaluation of the anti-tumor efficacy of CD19 CAR-T (CD3z-41BB) cells (data were obtained from four cohorts of embryos within a single experimental run): (**a**) Tumor growth evaluation through tumor mass weight (mean value ± SEM) in different groups at EDD18 (*n* = 16–20 per group); (**b**) metastasis evaluation through relative *Alu* sequence expression (mean value ± SEM) in the lower CAM in different groups measured by qPCR at EDD18, with an arbitrary value of biomarker expression at 1 for the non-treated (negative control) group (*n* = 8 per group); (**c**) angiogenesis evaluation by counting vessels arriving at the tumor in different groups at EDD16 (*n* = 10 per group); (**d**) representative photos of vessels perfusing the tumors (scale bar = 5 mm). Statistics were calculated through a one-way ANOVA analysis (with Tukey’s multiple comparison post-tests for all pairwise group comparisons): no stars, no statistical difference (*p* value > 0.05); two stars (**), 0.01 ≥ *p* value > 0.001; three stars (***), 0.001 ≥ *p* value > 0.0001; four stars (****), 0.0001 ≥ *p* value.

To validate the CAM assay for studying CAR-T cells, the second CAR-T-cell agent—a CAR-T (3Z-41BB) cell expressing a different second-generation CAR construct—was continuously evaluated. Based on results obtained with CAR-T (CD3z-CD28) cells, the regimen used for CAR-T (3Z-41BB) cells was modified: dosing at 0.02 million cells per egg, i.e., an effector-to-tumor ratio (E:T) of 0.2:1, either once at EDD11, or twice at EDD11 and EDD14. Furthermore, the importance of the active intracellular-signaling domain was shown in the previous test—thus, the “Raji + CAR-T (-) E11/E14” control group was not necessary. For this reason, we kept only the two remaining controls: (I) the negative control “Raji, NO CAR-T”, where embryos were grafted with Raji cells but did not receive any treatment; (II) “NO Raji, CAR-T E11/E14”, where embryos were not grafted with Raji cells at EDD9 but were administered CAR-T (3Z-41BB) cells at EDD11 and EDD14, was used for interpreting the CAR-T cells’ anti-tumor efficacy. The efficacy of CAR-T (3Z-41BB) cells was evaluated through tumor growth, metastasis, and angiogenesis. Figure 6a shows a tumor growth regression tendency in the group treated once with CAR-T (3Z-41BB) cells at EDD11 (“Raji + CAR-T E11”: a regression of 17,83%, *p* = 0.5707), when compared to the “Raji, NO CAR-T” group; no change is found in the group treated twice (“Raji + CAR-T E11/E14”). Figure 6b presents the metastatic invasion in the lower CAM in the different groups. When compared to the “Raji, NO CAR-T” group, a significant metastatic regression was revealed in both groups treated with CAR-T cells (“Raji + CAR-T E11”, a regression of 94.2%, **** *p* < 0.0001; “Raji + CAR-T E11/E14”, a regression of 68.04%, *** *p* = 0.001); the higher regression percentage was found in the “Raji + CAR-T E11” group. Figure 6c presents the results of the angiogenesis analysis, which demonstrates a significant inhibition of angiogenesis in the “Raji + CAR-T E11” group when compared to the “Raji, NO CAR-T” group (a regression of 33.94%, *** *p* = 0.0003), while the change found in the “Raji + CAR-T E11/E14” group was not evident (a regression of 7.98%, *p* = 0.5339). The representative photos of tumors surrounding vessels taken in different groups are shown in Figure 6d.

## 3. Discussion

Even though remarkable clinical responses to CAR-T-cell therapy have been reported in certain subsets of B-cell leukemia or lymphoma, several limitations to its efficacy against most solid tumors need to be addressed, including severe life-threatening toxicities, antigen escape, restricted trafficking and limited tumor infiltration, the impact of an immunosuppressive microenvironment, and others. The development of CAR-T-cell therapies requires a relevant in vivo model that complies with current 3Rs principles. In this context, we studied two CD19-targeting CAR-T-cell agents provided by Allogenica (France) against a human lymphoma model derived from the Raji cell line, to evaluate the chicken CAM assay as an alternative in vivo model for CAR-T-cell therapy testing.

CD19 is a B-cell lineage-specific antigen expressed on the cell surface of most B-cell lymphomas. CD19 expression covers the entire spectrum of early B-cell genesis and maturation, and its potential has been shown clinically for the treatment of B-cell lymphomas [26], as well as in the treatment of autoimmune diseases. Allogenica (France), which develops advanced, universal T-cell therapies, has provided two cell agents expressing well-characterized benchmark CD19-CARs (based on the FMC-63 scFv), used in similar forms in various previous studies. The cells tested in this study are classified as the second generation of CAR-T cells, expressing an extracellular antigen-recognizing domain combined with two intracellular domains: CD3z and an additional costimulatory domain (e.g., CD28 or 4-1BB) (Figure S1). The constant expression of CD19 by the Raji lymphoma cell line was confirmed by flow cytometry before the study. The in ovo CDX (cell-derived xenograft) derived from the Raji cell line resulted in consistent and homogeneous tumor growth (Figure 1). These results ensure the relevance of the tumor model used in this work.

In the first in ovo tumor challenge study, CAR-T (CD3z-CD28) cells were tested with a single dose of 1 × 10^5^ cells per embryo at each administration, corresponding to an effector-to-tumor ratio (E:T) of 1:1 and 1.67 million cells/kg, either once at EDD11, or twice at EDD11 and EDD14. This dose was selected based on our in vitro toxicity data (Figure S1) and clinical observations indicating optimal efficacy of anti-CD19 CAR-T-cell therapy at doses between 1 and 4.9 million cells/kg [27]. The regimen consisting of a single administration of CAR-T cells at EDD14 was not implemented, owing to established knowledge of chicken embryonic immune development [23,28] and our prior experience with anti-cancer drug testing in the CAM model [20,29,30,31]. Indeed, the first treatment in ovo should be administered before EDD14 because (1) the experimental window for in ovo CAM assays runs from EDD9 to EDD18, and without intervention, tumor burden is already substantial by EDD14, making it difficult to interpret anti-tumor efficacy if treatment begins only at this stage; (2) for immune-oncology therapeutics, in particular, earlier administration is advisable because a more mature embryonic immune system increases the likelihood of immune rejection against the therapeutic cells. However, to address the concern that observed effects were potentially attributable to an adaptive immune response against CAR-T cells, we included the “Raji + CAR-T (-) E11/E14” control group, in which embryos were grafted with Raji cells at EDD9 and treated with the control “CAR-T (-) cells”—expressing the CAR extracellular domain but lacking the intracellular-activation domain—at the same time points and doses. Because CAR-T (-) and CAR-T (CD3z-CD28) cells share the same antigenic profile, both would be expected to elicit a comparable adaptive immune response, if present. In addition, this control is relevant to confirm the necessity of a fully functional CAR construct (extracellular CAR, transmembrane region, and intracellular-signaling domain). Both tested regimens were well tolerated in ovo, and no abnormal embryo mortality was observed in any of the groups treated with CAR-T cells (Figure S2). Efficient tumor growth inhibition was observed in the group treated once with CAR-T (CD3z-CD28) cells at EDD11, but not in the group treated twice (Figure 2a). Moreover, two treatments with CAR-T (CD3z-CD28) cells led to an evident metastatic progression (Figure 2b). These observations drove us to further investigate the activation of the CAR-T cells as well as the intrinsic immune reaction of the chicken embryo.

First, the in ovo activation of CAR-T cells and their functionality in the tumor were investigated through the intra-tumoral gene expression of human IFNγ and human TNFα by RT-qPCR at EDD18. The expression of both cytokines, being major effector cytokines released by CAR-T cells after the recognition of tumor antigens, showed similar tendencies. Both were upregulated in the tumor after the administration of CAR-T cells, and two doses of CAR-T cells resulted in a higher level of gene expression (Figure 3). These findings suggest a good activation and functionality of CAR-T cells after administration in ovo.

Second, a relevant in vivo model for analyzing CAR-T cells should possess a tumor microenvironment (TME), allowing the study of the endogenous immune reactions that align with the clinical observations, such as cytokine release syndrome (CRS). CRS is difficult to predict as it is not merely the consequence of a binary tumor–CAR-T-cell interaction, but also the result of a multicellular network including endogenous reactions. IL-6 is one of the most elevated endogenous cytokines observed during CAR-T-cell-induced CRS [32,33], for which macrophages and monocyte lineage cells are the major source. Giavridis T et al. showed that several endogenous cell populations succeeded in infiltrating the tumor site in a CD19+ lymphoma xenograft model, including dendritic cells, monocytes, and macrophages, with macrophages vastly outnumbering the other cell types [34]. Therefore, to investigate the activation of endogenous immune responses, we first checked for the infiltration of embryo macrophages in the tumor and the endogenous IL-6 expression at the tumor site. The observations for both agents were correlated, and an evident increase in MMD (the macrophage-representing marker) expression was found in the group treated twice with CD19 CAR-T cells (Figure 4a), with a significant upregulation of IL-6 at the tumor site in this group (Figure 4b). At the same time, IL-8, another cytokine involved in CRS clinically, was also found to have a moderate increase in the same groups (Figure 4c). These findings indicate a stronger inflammatory response following two doses of CAR-T cells, as evidenced by the increased leukocyte infiltration observed in H&E-stained histological sections (Figure 5a,b). Immunohistochemistry (IHC) analysis of specific immune cells could not be performed due to the limited availability of chicken-specific antibodies. IL-6 is described as an important cytokine in tumor initiation and progression. The effects of IL-6 can be direct on tumor cells, as well as indirect through the promotion of angiogenesis, invasiveness, and metastasis [35,36,37]. Meanwhile, IFNγ is a potent pro-inflammatory agent and plays a dual role in tumor progression. Despite its anti-tumor effects, IFNγ can also induce the expression of PD-L1 and indoleamine 2,3-dioxygenase (IDO) and can therefore contribute to the tumor’s escape from immune surveillance and allow tumor progression [38]. Because of these concerns, the blockade of IL-6/IFNγ has been considered to prevent the side effects induced by CAR-T cells [32,39]. Zhang et al. presented IL-6/IFNγ double knockdown as a promising strategy for improving this therapy [40]. Given the high activity of IFNγ-expressing CAR-T cells and the strong inflammatory responses observed after two doses, the interplay between CAR-T-derived inflammatory factors and those produced by endogenous immune cells may help explain the limited impact on primary tumor growth and the marked increase in metastatic progression in this group. Indeed, inflammation is a major component of the TME that promotes metastatic progression, but it is not the only one. Other factors in the TME that provide biochemical, cellular, and structural cues—such as hypoxia, metabolic stress, interactions with stromal cells like cancer-associated fibroblasts, excessive cytokine-activated pathways including epithelial–mesenchymal transition (EMT), angiogenesis, and immune evasion, among others—could also enhance tumor cell invasion and dissemination [41,42,43,44,45,46]. Although these aspects were not investigated in this work, future studies may provide additional insights into their roles.

Beyond the pro-inflammatory responses, we observed the activation of endogenous T cells and their engagement in the tumor challenge (Figure 4d–f). Recent research indicates that, in the context of adoptive cell transfer, CAR-T-cell-based strategies that induce endogenous T-cell responses may help overcome antigen escape [47]. Our findings highlight the relevance of the chicken embryo model, and, in particular, the CAM assay, for the preclinical development of CAR-T therapies.

Building on findings observed with CAR-T (CD3z-CD28) cells, we next evaluated another CAR-T cell construct, CAR-T (3Z-41BB), at a five-fold lower dose, to assess its efficacy while limiting inflammatory effects. Although the tumor growth restriction achieved by CAR-T (3Z-41BB) cells under this condition was moderate, the metastasis promotion seen at the higher CAR-T dose was reversed, and a significant metastatic regression was observed, consistent with an evident inhibition of angiogenesis in the primary tumor. Once again, a single CAR-T-cell dose yielded better results than two doses (Figure 6). These results suggest that defining an appropriate dosing regimen that balances the therapeutic efficacy of CAR-T cells with the associated side effects—such as inflammation and toxicity—is essential for optimizing CAR-T-cell therapy. Further analyses of CAR-T-cell persistence, viability, and expansion in ovo would help address this need. In addition, modulating endogenous immune responses, particularly inflammatory pathways, represents a promising strategy to improve CAR-T-cell therapy outcomes, and combination approaches, for example, with anti-inflammatory agents, may help mitigate the adverse effects.

## 4. Materials and Methods

### 4.1. CD19-Targeting CAR-T Construction

Allogenica France provided two CD19-targeting research CAR-T-cell agents (CAR-T (CD3z-CD28) and CAR-T (3Z-41BB)) (Figure S1). CARs were generated by fusing an anti-CD19 scFv(FMC63) to a CD8 hinge and TM, followed by either a CD28 costimulatory domain or a 41BB costimulatory domain and a CD3z activation region. To allow facilitated tracking, an eGFP was fused in line with the CAR following a 2A site. The CARs were placed in an SFFV-promoter-driven, 3rd-gen lentiviral vector backbone and produced as VSVG-pseudotyped vectors. Primary T cells were isolated from PBMC, activated with CD3/CD28 Beads (Dynabeads, Life Tech, Saint-Aubin, France), transduced to 40–60%, and expanded for one week before freezing at −150 °C.

### 4.2. Tumor Cell Culture

The human Raji tumor cell line was obtained from the American Type Culture Collection (ATCC). Raji cells were cultivated in RPMI 1640 medium supplemented with 10% fetal bovine serum (FBS) and penicillin (100 units/mL)/streptomycin (100 μg/mL) (Sigma-Aldrich, Saint-Quentin-Fallavier, France). Cell culture was maintained at 37 °C in a 5% CO_2_ environment.

### 4.3. FACS Analysis of CD19 Expression by Raji Cells

Raji cells were stained with anti-human CD19-PE (ThermoFisher Scientific, Illkirch-Graffenstaden, France) according to the manufacturer’s instructions. All data were acquired with an Attune NXT flow cytometer using Attune Cytometric Software (version 5.3.0, ThermoFisher Scientific, Illkirch-Graffenstaden, France) and analyzed by FCS Express V7 software (version 7.18.0025, De Novo Software, Pasadena, CA, USA).

### 4.4. Chicken Chorioallantoic Membrane Assay (CAM) Assay

Fertilized Novo BT chicken eggs were obtained from Couvoir Hubert, Guilberville, France. Eggs were incubated at 37.5 °C with 50% relative humidity for 9 days. At EDD9, the CAM was dropped down by drilling a small hole through the eggshell into the air sac, allowing the air to escape, and a 1 cm^2^ window was cut in the eggshell above the CAM. Tumor cells were detached with trypsin, washed with complete medium, and suspended in graft medium. An inoculum of 1 × 10^5^ cells in 50 µL complete culture medium containing 50% Matrigel was added onto the CAM of each embryo. Then, the hole was sealed with adhesive tape, and the egg was placed in the incubator in a stationary position. Living eggs were randomized into groups before CAR-T-cell treatment (described in the next paragraph). Embryonic viability was checked daily. Any dead eggs were removed and disposed of appropriately. The CAM assay ended at EDD18.

### 4.5. In Ovo Tumor Treatment

At 24 h after tumor cell engraftment (at EDD10), tumors began to be detectable. CAR-T cells were administered on CAM at EDD11. Then, 24 h before in ovo administration, CAR-T cells were dissolved in RPMI supplemented with 20% FBS, centrifuged at 1000 rpm for 10 min, then resuspended in complete medium (RPMI supplemented with 10% FBS and 1% penicillin/streptomycin + hIL2 10 ng/mL (Sigma-Aldrich, Saint-Quentin-Fallavier, France)) and incubated at 37 °C. CD19 CAR-T (CD3z-CD28) cells were dosed at 1 × 10^5^ cells per administration, per embryo. CD19 CAR-T (CD3z-4-1B) cells were dosed at 0.2 × 10^5^ cells per administration, per embryo. CAR-T-cell treatment was performed either once at EDD11 or twice at EDD11 and EDD14. Control groups included: (I) Raji-grafted embryos without CAR-T-cell treatment; (II) Raji-grafted embryos treated with the control CAR-T cells (without active intracellular-signaling domain), twice at EDD11 and EDD14; (III) non-Raji-grafted embryos treated with CAR-T cells, twice at EDD11 and EDD14.

### 4.6. Tumor Harvest

At EDD18, living embryos were sacrificed; the upper portion of the CAM (with tumor) was removed, washed with PBS buffer, and directly transferred into 4% paraformaldehyde (PFA) (Sigma-Aldrich, Saint-Quentin-Fallavier, France) (fixation for 48 h at 4 °C). Tumors were then carefully cut away from the normal CAM tissue and weighed for a quantitative evaluation of tumor growth. Fixed tumor samples were preserved in 70% ethanol for later histology analyses.

### 4.7. Quantitative Evaluation of Metastatic Invasion by qPCR

Evaluation of the metastatic invasion was performed by detecting human *Alu* sequences in genomic DNA extracted from the lower CAM (opposed to the grafting site). This method is well established and widely reported in the literature for CAM assay applications [48,49,50]. Briefly, among the living embryos from which the tumor was collected for tumor growth analysis, a 1 cm^2^ portion of the lower CAM was collected from 8 embryos per group. Genomic DNA was extracted using the NucleoMag 96 Tissue kit (Macherey-Nagel, Hoerdt, France) on the Kingfisher Duo Prime System (ThermoFisher Scientific, Illkirch-Graffenstaden, France). gDNA purity was validated by measuring the A260/A280 ratio and A260/A230 ratio using a Multiscan GO (ThermoFisher Scientific, Illkirch-Graffenstaden, France) equipped with IDrop system (ThermoFisher Scientific, Illkirch-Graffenstaden, France). For qPCR, 50 ng of genomic DNA inputs was used per reaction, with each sample being performed in duplicate. Pure chicken DNA was used to determine the limit of detection, and water served as a no-template control. Hydrolysis probe-based primer pairs used to detect human *Alu* sequences were used as previously described and validated by Funakoshi et al. [51]. Linearity and amplification efficacy were determined using a standard curve generated from serial dilutions of pure human genomic DNA. Primer pairs were used at a final concentration of 25 nM in the qPCR reaction. Chicken GAPDH served as housekeeping gene to normalize variability in chicken DNA content across samples. All primers were synthesized by Bio-Rad (Marnes-la-Coquette, France). qPCR conditions consisted of an initial denaturation step at 95 °C for 10 min, followed by 40 cycles of 95 °C for 15 s, 56 °C for 45 s, and 72 °C for 30 s. Cq values were obtained using the CFX96 Touch system (Bio-Rad, France), using default settings. Relative quantities (ddCq) were calculated using the Bio-Rad^®^ CFX Maestro software (Version 5.0.021.0616, Bio-Rad, Marnes-la-Coquette, France), assigning an arbitrary metastasis value of 1 to the non-treated (negative control) group.

### 4.8. Quantitative Evaluation of Immune Cell Infiltration by RT-qPCR

Among the tumors collected at EDD18, 8 tumors per group were used to evaluate the infiltration of immune cells. Each tumor was cut to a small size (<0.5 cm^3^) and kept in 5 volumes of RNA Safeguard (BioFlux, Labtech France, Palaiseau, France) solution at 4 °C. Tumor tissue was submitted to lysis, and subsequently, RNA was extracted using the MagMAXTM mirVana kit (ThermoFisher Scientific, Illkirch-Graffenstaden, France). RNA concentration was determined through absorbance at 260 nm using a Multiscan GO (ThermoFisher Scientific, Illkirch-Graffenstaden, France) with microDrop system (ThermoFisher Scientific, Illkirch-Graffenstaden, France). RNA purity was validated by measuring the A260/A280 ratio and A260/A230 ratio. The reverse transcription of extracted RNA was performed using the iScript Explore RT and Preamp kit (Bio-Rad, Marnes-la-Coquette, France). cDNA was further used for qPCR analysis (PrimePCR™ Probe Assay and iQ Multiplex Powermix, Bio-Rad, Marnes-la-Coquette, France) with specific primers for human IFNα and TNFα sequences and chicken MMD, IL-6, IL-8, CD3, CD8, and CD4 sequences. Human GAPDH served as housekeeping gene to normalize variability in human DNA content across samples. All primers were synthesized by Bio-Rad (Marnes-la-Coquette, France). Each sample’s Cq and relative marker gene expression level were directly calculated and managed by the Bio-Rad^®^ CFX Maestro software (Bio-Rad, Marnes-la-Coquette, France). qPCR conditions consisted of one denaturation step at 95 °C for 2 min, followed by 40 cycles at 95 °C for 5 s and 60 °C for 30 s. The Cq values were calculated using the CFX96 Touch system (Bio-Rad, Marnes-la-Coquette, France), using the default settings. Relative quantities (ddCq) were calculated directly on the MAESTRO software (Version 5.0.021.0616, Bio-Rad, Marnes-la-Coquette, France), with an arbitrary biomarker expression value of 1 for the non-treated (the negative control) group.

### 4.9. Histochemistry Analyses

The 4% PFA fixed tumor samples were rinsed with phosphate-buffered saline (PBS) and put in 70% ethanol. Each tumor sample was embedded in paraffin and sectioned at 5 µm. Sections were stained with hematoxylin and eosin (H&E) (ScyTek, Inc., Logan, UT, USA). Pictures were taken using a Zeiss microscope (Axio Imager M2, Carl Zeiss Microscopy GmbH, Jena, Germany) at a magnification of 20×, and the microscope’s camera (AxioCam Mrc5, Carl Zeiss Microscopy GmbH, Jena, Germany). Image analyses were performed on 10 fields per section. A semi-quantitative quantification was performed using a scoring scale as follows: Mitosis Index: Grade 0 = 0 mitotic cell, Grade 1 = 1–2 mitotic cells, Grade 2 = 2–3 mitotic cells, Grade 3 = > 3 mitotic cells; Inflammation: Grade 0 = scant or absent inflammatory cells, Grade 1 = inflammatory cells obviously present but markedly less than other cells, Grade 2 = inflammatory cells roughly equal to tumor cells; Grade 3 = predominantly inflammatory cells; Necrosis: Grade 0 = no necrosis, Grade 1 = small foci of necrosis or widespread single cell necrosis that required careful perusal of the section, Grade 2 = obvious presence of necrosis, but in <50% of the field, Grade 3 = necrosis in >50% < 75% of the field, Grade 4 = necrosis in >75% < 90% of the field, Grade 5 = necrosis in 90% of the field.

### 4.10. Angiogenesis Analyses

Images of the tumors grown in ovo were captured at EDD16 through a stereomicroscope (Olympus SZX16, Olympus Corporation, Hachioji, Tokyo, Japan). Angiogenesis was then analyzed through the manual counting of blood vessels perfusing the tumor. Three independent counts were performed. Tumor vessel counting was performed for 10 eggs per group.

### 4.11. Statistical Analysis and Significance

All quantitative data were analyzed with the specialized computer software Prism^®^ (GraphPad Software, version 10.6.1). For comparisons between two groups, an unpaired *t*-test was applied. For comparisons between more than two groups, a one-way ANOVA analysis (with Tukey’s multiple comparison post-tests between each pair of groups) was performed. Embryo survival was presented using a Kaplan–Meier curve, and a Log-rank (Mantel–Cox) test was used to interpret the treatment-induced embryo toxicity. Outliers were identified by the ROUT test. For all analyses, the statistical difference between groups was indicated on graphs with stars: No stars: no statistical difference (*p* value > 0.05); one star (*): 0.05 ≥ *p* value > 0.01; two stars (**): 0.01 ≥ *p* value > 0.001; three stars (***): 0.001 ≥ *p* value > 0.0001; four stars (****): 0.0001 ≥ *p* value.

## 5. Conclusions

In this work, the anti-tumor efficacy of CD19-targeted CAR-T-cell agents provided by Allogenica (France) against human lymphoma was confirmed in vivo using the chicken CAM assay. The efficacy of CAR-T cells was shown directly on the tumors, but also on the TME and the entire organism. Unlike humanized mouse models, chicken embryos possess an active, native immune system. This allows monitoring endogenous immune response activation and its participation during the tumor challenge. Furthermore, the use of the CAM assay for testing human PD-1/PD-L1 inhibitors has been previously reported [20]; various combination regimens and immune modulation strategies, such as PD-1/PD-L1 checkpoint blockade, could also be considered. These results provide a solid foundation for future investigations to help uncover key human cell factors interacting with the chicken immune system, shedding light on the mechanism driving the observed responses. Overall, the CAM assay represents a relevant in vivo, 3Rs-compliant NAM well-suited to the current needs of preclinical research for improving CAR-T therapy.

## Data Availability

The original contributions presented in this study are included in the article/Supplementary Materials. Further inquiries can be directed to the corresponding author.

## Supplementary Materials

The following supporting information can be downloaded at: https://www.mdpi.com/article/10.3390/ijms27020795/s1.

## Abbreviations

The following abbreviations are used in this manuscript:

CAR	Chimeric antigen receptor
CAM	Chicken ChorioAllantoic Membrane
CD	Cluster of differentiation
CDX	Cell-Derived Xenograft)
CRS	Cytokine Release Syndrome
EDD	Embryonic Development Day
FDA	Food and Drug Administration
IFNγ	Interferon gamma
IL	Interleukin
MHC	Major Histocompatibility Complex
MMD	Macrophage-representing marker
NAMs	New Approach Methodologies
3Rs	Replacement, Reduction, and Refinement
SEM	Standard Error of the Mean
TCR	T-Cell Receptor
TME	Tumor MicroEnvironment
TNFα	Tumor Necrosis Factor alpha
