# Supplementary material for: An Innovative In Vivo Model for CAR-T-Cell Therapy Development: Efficacy Evaluation of CD19-Targeting CAR-T Cells on Human Lymphoma, Using the Chicken CAM Assay
Source: Int J Mol Sci. 2026 Jan 13;27(2):795. doi: 10.3390/ijms27020795 (PMC12841510; doi:10.3390/ijms27020795)
Supplement: Supplementary file 1 [file ijms-27-00795-s001.zip › ijms-4023995-supplementary.pdf]

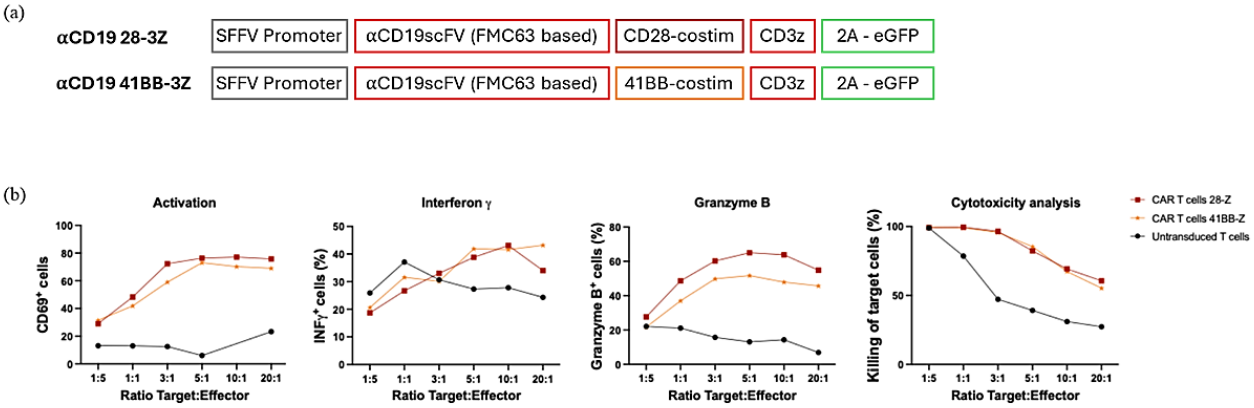


**Figure S1.** CD19 specific CAR-T (CD3z-CD28) & CAR-T (3Z-41BB) cells construction and characterization: (a) CD19-specific CARs were constructed by cloning FMC63-based 2nd generation CARs into a 3rd generation, self-inactivating, lentiviral vector research-backbone. The CARs were expressed via a strong Spleen Focus Forming Virus (SFFV) promoter and packaged into a VSVG-pseudo-typed 3rd gen. lentiviral vector. An overview of the major elements is shown. (b) CAR-T cells were generated by activating primary human T cells from healthy donor PBMC with an adapted amount of lentiviral vector to obtain 40-60% transduction. Cells were expanded for 7 days before being characterized in co-culture with CD19 expressing target cells (NALM-6) at different ratios of CAR-T cells to target tumor cells. As a control, we used non-transduced T cells from the same donor (black lines). Both vectors demonstrated robust functionality in vitro by dose dependent activation (via CD69), interferon γ secretion and granzyme B upregulation, as well as proficient killing of target cells. No pronounced differences in cytotoxicity were observed in vitro between both CARs using a standard killing assay of Nalm-6 target cells. Cytotoxic efficacy is presented as percent killing of target cells, where 100% killing corresponds to 0% viable target cells, and 0% killing indicates that all target cells remained viable relative to the "No–T cell" control condition.

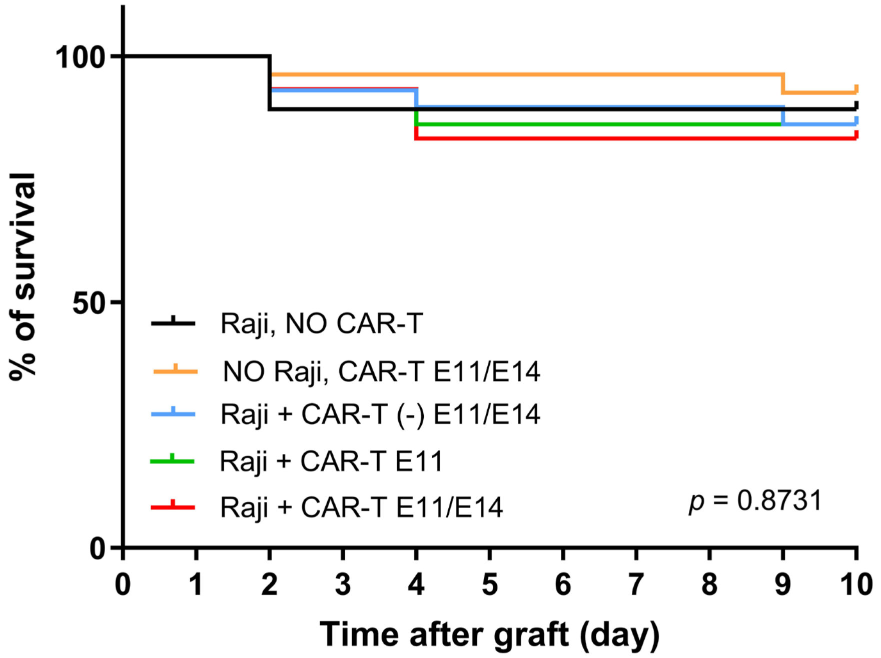


**Figure S2.** In ovo tolerability of CAR-T (CD3z-CD28) cell treatment: the Kaplan-Meier curve showed the survival rate for all groups during the efficacy study with CD19 CAR-T (CD3z-CD28) cells (from graft day EDD9 to collection day EDD18); no evident embryo mortality was observed in any experimental group, when compared with the negative control group.
